# Increasing γ-Aminobutyric Acid Content in Vegetable Soybeans via High-Pressure Processing and Efficacy of Their Antidepressant-Like Activity in Mice

**DOI:** 10.3390/foods9111673

**Published:** 2020-11-16

**Authors:** Sz-Jie Wu, Chia-Yu Chang, Yen-Ting Lai, Yuan-Tay Shyu

**Affiliations:** Department of Horticulture and Landscape Architecture, National Taiwan University, No. 1, Section 4, Roosevelt Road, Taipei 10617, Taiwan; chiehwu@ntu.edu.tw (S.-J.W.); r06628211@ntu.edu.tw (C.-Y.C.); r04628211@ntu.edu.tw (Y.-T.L.)

**Keywords:** γ-aminobutyric acid, vegetable soybean, high-pressure processing, unpredictable chronic mild stress

## Abstract

This study applied high-pressure processing (HPP) technology to enrich the gamma aminobutyric acid (GABA) content in vegetable soybeans and evaluated its antidepressant efficacy on mice, with depression induced by the unpredictable chronic mild stress (UCMS) model. The optimal conditions for HPP, storage time, and storage temperature, as well as antidepressant-like effects of vegetable soybeans, were evaluated and discussed. HPP could effectively and significantly increase GABA content in soybean, with optimum conditions at 200 MPa. The GABA content in the whole vegetable soybean was 436.05 mg/100 g. In mice animal tests, the tail suspension test (TST) showed that the immobility time of the GABA group was significantly shorter than that of the control group. The total travel distance in the open field test (OFT) showed that depressed mice fed with the GABA feed exhibited exploratory behavior. The GABA group showed a significantly higher degree of sucrose preference than the control group. Both results indicate that the GABA feed could effectively alleviate depressive symptomatology. Regarding biochemical parameters, the fecal and serum corticosterone (CORT) levels in the control group increased to 104.86 pg/mg after the onset of depression. In contrast, the fecal CORT level in the GABA group was significantly reduced to 23.98 pg/mg and was comparable to that in the control group (33.38 pg/mg). Reduced serum CORT level in the GABA group suggests an improvement in depressive symptomatology. The serotonin concentration was maintained in the GABA group after the induction of depression, suggesting its preventive activity. The HPP GABA-enriched soybeans exerted modulatory effects on the behaviors of depressed mice and displayed a potential for commercialization.

## 1. Introduction

Vegetable soybean (*Glycine max* (L.) Merr.) has a high density of nutrient-rich macromolecules and is popularly consumed as a vegetable or snack especially in Asian countries. In addition to a 13% protein content, vegetable soybean is a complete protein source as it contains all nine essential amino acids required by the human body. It is also rich in unsaturated fatty acids and vitamins [1,2]. To a lesser degree, vegetable soybeans contain trace amounts of raffinose that can easily cause flatulence. Furthermore, vegetable soybeans contain several functional ingredients, such as flavones, saponins, lecithin, and γ-aminobutyric acid (GABA), which increases its added value [3]. Many foods made from grain soybeans are fermented, but the fermentation of protein-rich soybeans may produce biogenic amines that may cause human poisoning [4]. Therefore, some novel processing technologies and product forms from various soybeans were investigated and developed to avoid potential undesired ingredients and to increase functional components. For example, Kim et al. [5] reported that puffing is an efficient process to increase antioxidant activity, total reducing capacity, and flavonoid contents of black soybeans.

GABA is a non-protein amino acid present in small amounts in plants, where a typical GABA concentration ranges from 0.31–56.7 mg/100 g. When encountering adversity, plants produce a considerable amount of GABA to cope with it. For example, when plants are subjected to an unfavorable environment, such as drought, high salinity, cold/high temperature, and an anaerobic environment, GABA is readily accumulated [6,7]. GABA, a naturally occurring amino acid in the human body, is regarded as the major inhibitory neurotransmitter in the mammalian central nervous system and plays a vital role in maintaining mental health. [8]. Thus, GABA can affect several physiological processes, including accelerating brain protein synthesis, increasing growth hormone concentration, reducing high blood pressure, controlling diabetes, and promoting diuresis [9]. In addition, it can also exert a calming effect and may be used to treat insomnia, depression, and post-traumatic stress syndrome (PTSD). Previous studies have demonstrated that GABA contributes to the management of stress, pain, and anxiety [10,11,12,13,14]. Injection of 100 and 150 μg/10μL of physiological saline of GABA into mice has been shown to improve their ability in discrimination learning [15]. It has been reported that menopausal women taking GABA experienced a tranquilizing effect and relief of the aforementioned symptoms [16]. A study by Abdou et al. [17] demonstrated that acrophobic subjects exposed to high altitudes who consumed 100 mg of GABA displayed increased alpha-brain waves and reduced immunoglobulin A levels, which promoted relaxation and diminished anxiety.

Current GABA production methods are represented by the plant-enrichment method and by biosynthesis via microbial fermentation. In the plant-enrichment method, GABA is produced by accumulating under both biotic and abiotic stress conditions; this process is termed GABA enrichment [18]. The GABA contents in the seeds of grains or beans can be increased through germination. The nutrients contained in the seeds are enzymatically hydrolyzed during germination, consequently increasing the concentration of certain nutrients such as vitamins, monosaccharides, and amino acids [19,20]. Mung beans exhibit the highest GABA content of 0.8068 g/kg at 24 h after germination, which is 6.09-fold that in non-germinated beans [21]. Mechanical and cold stimulation can increase the GABA contents of soybeans by 20- to 40-fold [22]. A hypoxic environment can cause the GABA concentration of rice seedlings to reach 8 μmol/g fresh weight [23]. Moreover, GABA can be produced by bacteria and fungi, and fermentation conditions are also essential factors affecting GABA production. Currently, GABA is primarily produced by lactic acid bacteria (LAB) via fermentation; LAB has been isolated from several fermented foods, including kimchi, fresh milk, distiller’s grains, and blackberry juice. Anaerobic fermentation by *Rhizopus microspores* var. *oligosporus* IFO 32,002 and IFO 32,003 using soybean as a substrate is a rapid and high-yield production method that can produce 1.74 g/100 g of GABA [24,25]. Tofalo et al. [26] suggested that carefully selected autochthonous amine-negative and amine-oxidizing LAB could reduce butyric acid (BA) accumulation in traditionally-made cheese. Control of milk quality, ripening time, and coagulant type could increase GABA synthesis in dairy products. Recently, 50 *Kluyveromyces marxianus* strains isolated from different dairy products were tested for GABA production, and the genes involved in GABA catabolism and anabolism were thoroughly explored [27]. Cui et al. [28] systematically reviewed the biosynthesis pathway, the involved key enzymes, and the production enhancement strategies of GABA-producing lactic acid bacteria, and suggested that advances of synthetic biology and genetic engineering can improve the production of GABA by LAB.

HPP (high hydrostatic pressure processing) can generate a pressure of 100–1200 MPa through fluid compression. HPP technology can be used to preserve food, reduce microbial content, extend shelf life, alter cellular or tissue structures, and activate or inactivate enzymes in plant tissues, thereby modifying metabolism [29,30,31]. Previous studies have shown that glutamate decarboxylase (GAD) can be altered through HPP, which increases the GABA concentration in plant foods [32,33].

The vegetable soybean industry primarily relies on trade and is very important for the Taiwanese vegetable soybean industry. However, many products are discarded and wasted because they fail to meet the specifications of export commodities’ requirements such as appearance, shape, color, pod length, etc. Thus, searching for methods that serve to reuse these products is an important issue to address. As mentioned, GABA is a natural functional ingredient with antidepressant-like and antioxidant functions.

This study utilized HPP technology to enrich the functional ingredients of vegetable soybeans and sought an optimal approach and conditions for increasing GABA content. The changes in L-glutamate content in the GABA biosynthetic pathway are also discussed. GABA-enriched vegetable soybean seeds were ground into a powder and added to mouse feed, which was then fed to mice with depression induced by the unpredictable chronic mild stress (UCMS) model. The sucrose preference test (SPT), tail suspension test (TST), open field test (OFT), and biochemical parameter analyses were performed to assess the effects of the feed on depression in mice.

## 2. Materials and Methods

### 2.1. Materials

Fresh “Kaohsiung No. 9 vegetable soybeans” were purchased from Jiuruo fruit and vegetable markets in Pingtung, Taiwan. Part 1: The washed and dried vegetable soybean samples were aliquoted into polyethylene (PE) pouches and vacuum-sealed. They were processed with 100–600 MPa of HPP for 10 min at 25 °C (6 L, Bao Tou KeFa High Pressure Technology Co. Ltd., Inner Mongolia, China). The processed samples were stored at 4 °C, and sampling took place on days 0–5. Part 2: The washed and dried vegetable soybean samples were placed into PE pouches and vacuum-sealed. The samples were processed under optimal conditions (200 MPa, 10 min, 25 °C) for increasing GABA contents in vegetable soybeans. Next, the samples were placed in boiling water for 10 min, followed by freeze-drying, and further ground into a powder using a tissue homogenizer.

### 2.2. Methods

#### 2.2.1. High-Performance Liquid Chromatography (HPLC) Determination of GABA and L-Glutamate Contents

(1) Qualitative analysis of GABA standard

A 4 mg/mL stock solution was prepared in 70% ethanol and stored in a −20 °C freezer. The stock solution was diluted to a 0.5 mg/mL standard solution for derivatization. Qualitative analysis was performed by comparing the peak retention times of the samples and the standard in the respective HPLC chromatograms (Waters, Milford, MA, USA).

(2) Quantitative analysis of standards

The 4 mg/mL GABA and L-glutamate standard solutions were used, respectively, to prepare solutions with the following nine concentrations: 0.015625, 0.03125, 0.0625, 0.125, 0.25, 0.5, 1, 2, and 4 mg/mL. HPLC analysis was performed after derivatization. The experiment was performed twice. Standard curves were plotted using the concentration of the standards as the *x*-axis and the integrated peak areas of the standards as the *y*-axis. Standard curves were used as the bases for the quantitative analyses of the samples.

(3) Conversion formula for calculating concentration unit

Assuming the sample weight at extraction was 1 g, the final volume after extraction with 70% ethanol was 17.5 mL, the volume taken from the extract was 3.5 mL, the volume of the concentrated extract was 1 mL, the volume of the concentrated extract used for derivatization was 150 μL, and the final reconstitution volume of the eluted fraction was 1 mL. The GABA concentration obtained by fitting the integrated areas of the samples after HPLC separation into the standard curves was expressed in mg/mL.

#### 2.2.2. Extraction and HPLC Analysis of GABA

The ethanol extraction of GABA method was conducted with reference to a previous report [34]. One gram of sample powder was added to 7.5 mL of 70% EtOH, and the extraction was performed at 4 °C. The sample was centrifuged at 34,800× *g* at 4 °C for 20 min. The supernatants were concentrated to 1 mL using a vacuum reduced pressure concentrator. The Sample derivatization method was modified from previous reports by Chen [35] and Cohen and Strydom [36]. A total of 150 μL of the liquid extract was vacuum-dried (37 °C, 20 mmHg) and added to 20 μL of coupling reagent (methanol:water:triethylamine 2:2:1, *v*/*v*). After mixing, the solution was immediately subjected to vacuum drying for 30 min. A total of 30 μL of phenyl-isothiocyanate (PITC) reagent (methanol:water:triethylamine: PITC 7:1:1:1, *v*/*v*) was added, and the mixture was allowed to stand at 37 °C for 20 min. The PITC was subsequently removed by vacuum concentration. The PITC derivative was reconstituted in 1000 μL of solvent A. The HPLC analysis method was modified from a previous report by Chen [35]. Solvent A: each liter of solution contained 11.45 g of CH3COONa. The solution was added to 0.5 mL of Tris-acetate-EDTA (TAE), 65 mL of acetonitrile, and 1.07 mL of EDTA (0.1 g of EDTA dissolved in 100 mL of H_2_O). The final pH was adjusted to 5.30 with acetic acid. Solvent B: 60% (*v*/*v*) acetonitrile/H_2_O. HPLC analysis conditions: the chromatography column used was C18 reversed-phase column (C18 5U, 250 × 4.6 nm; Alltima^®^, College Park, GA, USA), the operating temperature was 40 °C, the detection wavelength was 254 nm, and the quantity of sample injected was 20 μL.

#### 2.2.3. Animal Experiments

In the animal experiments, the protocol and procedures employed were ethically reviewed and approved, and approval from the National Taiwan University office of research and development (NTU106-EL-00157) was given.

(1) Animal strains and experimental dosage

The four-week-old C57BL/6J male mice (*n* = 48) purchased from BioLASCO Taiwan Co., Ltd. (Taipei, Taiwan) were divided into four groups (*n* = 12/group), and were individually housed in temperature-and humidity-controlled cages. The recommended maximum intake of GABA in humans is 500 mg per day [37]. Using a 60 kg human adult as a reference and a 12.3-fold recommended intake for human per kilogram of body weight per day (mg/kg/d) as the dosage for mice, the recommended maximum daily intake for mice (average weight of about 25 g) was 102.5 mg/kg.

The preparation of GABA feed (enriched vegetable soybean + regular feed) was performed as follows: enriched vegetable soybeans processed by HPP treatment were freeze-dried and ground into a powder. The vegetable soybean powder was thoroughly mixed with the finely grounded regular feed LabDiet^®^ 5001, (LabDiet, St. Louis, MO, USA, composition and nutrient facts: https://www.labdiet.com). The mixture was then pressed into a mold and dried at 55 °C for 24 h to obtain a short cylindrical-shaped feed.

(2) Experimental groups

The mice were divided into the following four groups: UCMS group, GABA group, positive control group fed with regular feed mixed with commercial GABA tablets (BIOline Co., Taipei, Taiwan) that could help with sleeping; GABA content: 102.5 mg/kg, and control group. The experiment was conducted over a period of seven weeks. Treatment details of the groups were as follows:

UCMS group: fed with regular feed for the first three weeks; depression was induced at week 4, and the mice continued to receive regular feed in the last four weeks.

GABA group: fed with regular feed for the first three weeks; depression was induced at week 4, and the mice switched to the GABA feed for the last four weeks.

Positive control group: fed with regular feed for the first three weeks; depression was induced at week 4, and the mice switched to commercial GABA tablets for the last four weeks.

Control group: fed with regular feed for the full seven weeks; depression was not induced.

(3) Housing conditions of animals

The mice were individually housed in temperature- and humidity-controlled (23 ± 2 °C, 60 ± 10% Relative Humidity (%RH)) individually ventilated cages (IVC) with a 12/12 h light-dark cycle. The cage dimensions were 32 cm in length × 16.7 cm in width × 13.5 cm in height. The mice were housed for eight weeks, with the first week (week 0) as the quarantine period.

(a) The unpredictable chronic mild stress model

The animals were randomly stimulated with 6–8 different pressures for several hours. This treatment induces a depressed mood in experimental animals that is similar to that in humans. The induction method was based on a previous report by Farooq et al., with modifications [38]. This type of depression simulation model might aid our understanding of the pathological and physiological mechanisms involved in depressive disorders.

(b) Sucrose preference test

The SPT was conducted as described in Papp et al. [39] with modifications. The SPT was conducted once per week. After fasting for 6 h, a 1 h SPT was performed. Sucrose and pure water intakes within 1 h were measured. The degree of sucrose preference can be used as an indicator for anhedonia in animals. Thus, it was used to evaluate if the USCM induction was successful and served as the parameter for assessing the occurrence of depression in mice.

Formula for calculating sugar preference: ((intake of 1% sucrose)/(total liquid intake)) × 100%

(c) Open field test

OFT was conducted once per week. The experimental device was a white box of 41.3 cm in length, 41.3 cm in width, and 31.6 cm in height. The mice were placed in the box for 5 min, and software was used to analyze the total travel distance, number of rearings, number of grooming episodes, and number of defecations. The test was conducted with reference to the analysis method and experimental device described in Matsuo et al. [40] and Lopez et al. [41] with minor modifications.

(d) Tail suspension test

TST was conducted once a week. In TST, the time spent shaking the body to escape when the mouse was suspended in the air was evaluated. This record is similar to immobility in the forced-swim test. The test was performed with reference to the analysis method and experimental device described in Steru et al. [42] and Cryan et al. [43] with minor modifications. The device was a white box of 18 cm in length, 18 cm in width, and 25 cm in height. The mouse tail (1/3 from the tip) was affixed to a hole at the top of the box. The mouse was prevented from climbing on its own tail during the experiment. The entire experiment was video recorded, and the immobility time of the mice for 6 min was analyzed using the software.

(e) Biochemical parameter analyses

Corticosterone (CORT) was measured using the commercial StressXpress^®^ CORT EIA kit (StressMarq Biosciences Inc., Victoria, BC, Canada) according to the manufacturer’s protocol. Serotonin analysis was performed for the UCMS, GABA, and control groups. The analysis was performed using the commercial Serotonin ELISA Kit (StressMarq Biosciences Inc., Victoria, BC, Canada) according to the manufacturer’s protocol.

#### 2.2.4. Statistical Analysis

Data from part 1 of the experiment were expressed as mean ± standard deviation (SD). Analysis of variance (ANOVA) was performed using XLSTAT 2019 to test the significance of the differences in the experimental data (*p* < 0.05). The correlation coefficient calculation is based on the Pearson correlation coefficient, to observe whether there is a linear correlation between GABA and Glutamate content. In the statistical test of the significance of the correlation coefficient, the precondition of this study is *p* < 0.05. If the correlation coefficient is significant, it can be explained that the two variables are significantly correlated.

For part 2 of the experiment, one-way analysis of variance (One-Way ANOVA) was performed using statistical software IBM SPSS Statistics 23. In addition, Duncan’s multiple test was used to compare the differences between the means. The significant level was set at *p* < 0.05. The statistical results were expressed in lowercase Latin letters, and different lowercase letters represented different levels of significance.

## 3. Results and Discussion

### 3.1. Optimal Conditions for HPP of Vegetable Soybeans

**Figure 1:**
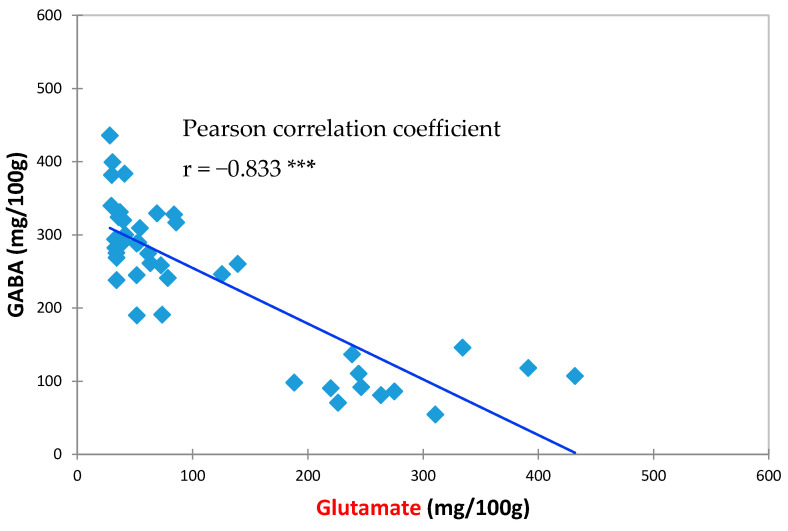
Correlation of GABA content with Glutamate content (scatter plot). *** *p* < 0.001, using Pearson correlation test.

Table 1 shows the results of different HPP treatments and storage days on the GABA content changes in vegetable soybean. Although there is a slight variation due to the plants’ nature, the GABA content can be stably retained for five days of storage after HPP treatment, while the GABA content of the control group without HPP treatment is dramatically reduced by 20.43% on the first day of storage. The highest GABA content of 436.05 mg/100 g was obtained with 200 MPa pressure and stored at 4 °C for 1 day (Table 1). The GAD activity is significantly increased when vegetable soybean was treated with 200 and 300 Mpa compared to those without HPP treatment, showing that the increase in GAD activity is related to the conversion of glutamate to GABA [44]. A previous study suggested that soybeans treated with 200 MPa of HPP had increased GABA contents during the preservation period of 1–3 d compared with soybeans without HPP treatment [32]. In the study by Sasagawa et al. [45] in which presoaked brown rice was subjected to a HPP treatment at 50–200 MPa for 10 min, the highest GABA content was obtained at 200 MPa, which is in accordance with the results of the present study. The data in Table 1 were analyzed by Pearson correlation analysis, showing that there is a highly negative correlation between GABA and Glutamate content (Figure 1, r = −0.833). In general, an r value between 0.75~1.0 or −0.75~−1.0 indicates its reliable relevance [46].

In the mechanism and effects of HPP treatment on phytochemicals in plants, Serment-Moreno et al. [47] pointed out that lower stress treatment can promote oxidative stress in plant tissues and induce secondary metabolites; however, excessive pressure treatment will rupture the cell membrane, increase the permeability of the cell wall, gradually stop the metabolic activity of plant cells, and significantly reduce cell viability. Moderate stress treatment will increase the rate of action and catalytic function of the GABA conversion enzyme in plants. The amount of GABA, GAD activity, and diamine oxidase (DAO) activity were all significantly increased compared to the control group in germinating fava bean under hypoxia [48]. Slightly acidic electrolyzed water (SAEW) treatment (pH 5.83, ACC of 20.3 mg/L) in germinating buckwheat seeds for 2 h can significantly increase GAD activity and increase the GABA content to 143.20 mg/100 g [49]. Under salinity (100 mM NaCl) treatment for 4–24 h, the accumulation of GABA and GAD activity showed an upward trend in leaves and roots of cultivated and wild tomato seedlings [50]. Therefore, this study further determined the changes in glutamate content in different HPP treatments.

### 3.2. Effects of HPP Treatment on GABA and Glutamate Contents in Vegetable Soybeans

GABA in plants is predominantly catalyzed from glutamate by GAD. This is an irreversible reaction that leads to the production of CO_2_ and GABA in the cytoplasm [51]. The results showed that there was a negative correlation between the amount of GABA and the amount of glutamate. The highest GABA content of 436.05 mg/100 g and the lowest glutamate content of 28.29 mg/100 g were obtained in the 200 MPa treated group. At 400 MPa, a moderate 288.46–329.55 mg/100 g of GABA and51.45–85.88 mg/100 g of glutamate can be obtained. At 600 MPa, there is the least GABA of 70.63–107.14 mg/100 g and the highest glutamate of 226.28–431.7 mg/100 g. The mechanism of GABA biosynthesis is complicated, regulated with a variety of enzymes and pathways. It is speculated that the HPP of 600 Mpa treatment may cause severe damage to those enzymes that convert glutamate to related metabolites, so there is a higher amount of glutamate accumulation. Ueno et al. [52] evaluated the effects of treatment of soybeans at 200 MPa for 10 min at 25 °C on L-glutamate concentration, GABA production, and their kinetics. Their results showed that the conversion of L-glutamate to GABA decreased with increasing initial L-glutamate concentration in soybeans. The K_m_ value in their study showed the affinity between L-glutamate and GAD, and the results revealed that the K_m_ value of HPP treated soybeans was significantly higher than that of untreated soybeans. These findings indicated that the affinity between L-glutamate and GAD was lower in the HPP treated compared to untreated soybeans. These results were consistent with the results of the present study. Another study showed that adding L-glutamate to the medium of germinated soybean embryos could lead to GABA accumulation [53]. Shigematsu et al. soaked brown rice grains in water and found that the optimal treatment time was 4 d, which could increase GABA content from the original 0.456 μmol/g·rice to 1.426 μmol/g·rice. HPP treatment at 200 MPa for 10 min significantly increased the GABA content to 1.734 μmol/g·rice [54]. Soybeans soaked in glutamate for two days and treated with HPP can achieve a high 4.2 μmol/g GABA content [32]. These previous studies all showed that glutamate is a key precursor and can be converted to GABA, and the glutamate content decreased when the amount of GABA increased. In this study, the trend of changes in GABA and glutamate content of vegetable soybeans under HPP treatment and in storage were similar to those that research has discovered in soaked soybeans and brown rice.

According to these speculations, HPP treatment can enhance the proteolysis and thereby increasing the supply of Glu, and HPP treatment can also change the apparent activity of GADs. Based on the evidence reported above, it can be speculated that an increase in GABA content leads to a reduction in L-glutamate content. In short, all showed an increasing trend with HPP up to 200–300 MPa, decreasing above a higher pressure level, and the contents varied in different storage days.

### 3.3. Animal Experiments

#### 3.3.1. Weight Changes during the Feeding Period

The mice were fed for eight weeks, and UCMS induction was conducted at week 4. At week 4, no significant differences in body weight were observed among the groups. At week 5, the weight of the UCMS group was lower than that of the control group. At weeks 6 and 7, the body weight was not significantly different between the UCMS and control groups. These findings were consistent with previous studies, indicating that UCMS induction exerts no significant impact on body weight in C57BL/6J mice [55]. UCMS induction for seven weeks in BALB/c ByJ mice did not lead to a significant difference in body weight when compared to that of the control mice [56]. Swiss and BALB/c mice exhibited no significant difference in body weight after six weeks of UCMS induction [57]. Weight change during the feeding period may affect the behavior of mice. However, the weight of all mice treated in the present study did not vary significantly; thus, the bodyweight factor can be ruled out.

#### 3.3.2. Degree of Sucrose Preference

In research tests, sucrose is frequently used as a reward for mice because it can activate dopaminergic neurons to releases dopamine [58]. However, rodents under stress easily lose interest in a sucrose solution and reduce sucrose intake. Therefore, testing for sucrose preference in mice can help evaluate the depression status induced by the experiment.

**Figure 2:**
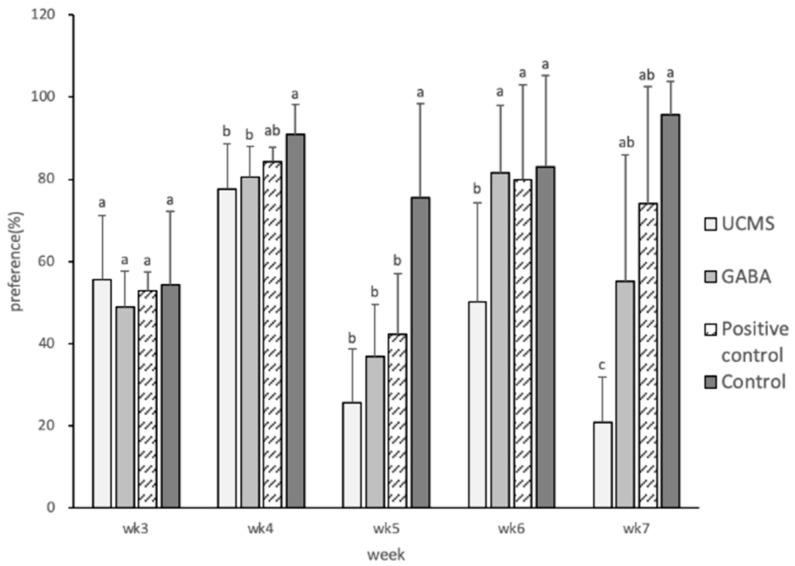
Effect of GABA feed made by vegetable soybean on sucrose preference in mice. Data are represented as mean ± SD. Group with different letter superscripts are significantly different (*p* < 0.05).

In the present study, depression was induced at week 4. The degree of sucrose preference in the control group was significantly higher than that in the UCMS and GABA groups. Symptoms of depression appeared starting from week 5. At week 6, the degree of sugar preference of the GABA and positive control groups increased significantly, reaching a similar degree of preference as that of the control group. However, the degree of preference was significantly lower in the UCMS group than in the other groups. These findings indicated that GABA feed could alleviate depression symptoms, leading to an increased proportion of sucrose intake by the mice (Figure 2). Deng et al. [59] employed the UCMS induction method in Institute of cancer research (ICR) mice for three weeks and found that the degree of sucrose preference was reduced by 22%, which was consistent with the results of the present study. In addition, the experiments conducted by Taksande et al. [60] showed that after four weeks of UCMS induction in Swiss mice the degree of sucrose preference was significantly lower on day 28 compared with day 14. The results of both experiments showed that the degree of sucrose preference of mice was reduced upon UCMS induction.

#### 3.3.3. Tail Suspension Test

**Figure 3:**
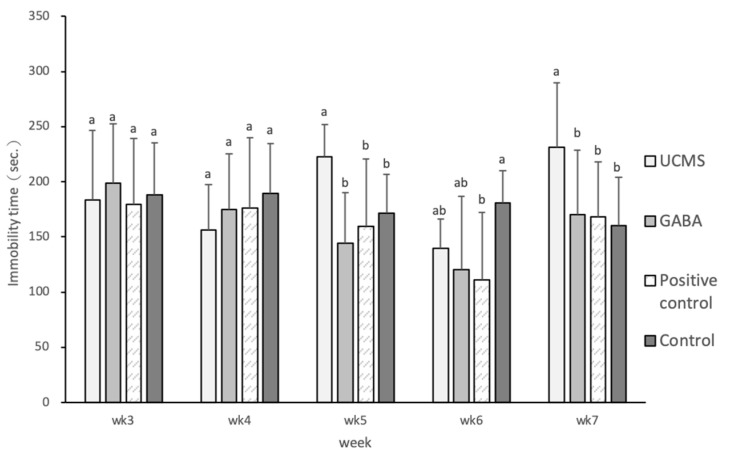
Effect of GABA feed by vegetable soybean on the immobility time of mice in tail suspension test (TST). Data are represented as mean ± SD. Group with different letter superscripts are significantly different (*p* < 0.05).

A mouse that is suspended by its tail and is unable to escape manifests initially an evasive behavior, followed by immobility. The typical duration of this test is 6 min, and immobility is used as a measure of despair. Depression was induced in mice starting at week 4. At weeks 5 and 7, the immobility time of mice in the UCMS group increased significantly, while that in mice of the GABA, positive control, and control groups had similar results. This observation showed that the GABA feed and commercial GABA tablets had a significant effect on alleviating depressive symptoms in mice (Figure 3). The study by Huang et al. [61] showed that C57BL/6 male mice subjected to UCMS induction had significantly longer immobility time than the mice not subjected to UCMS induction; the same results were also obtained in C57BL/6 female mice [62].

#### 3.3.4. Open Field Test

**Figure 4:**
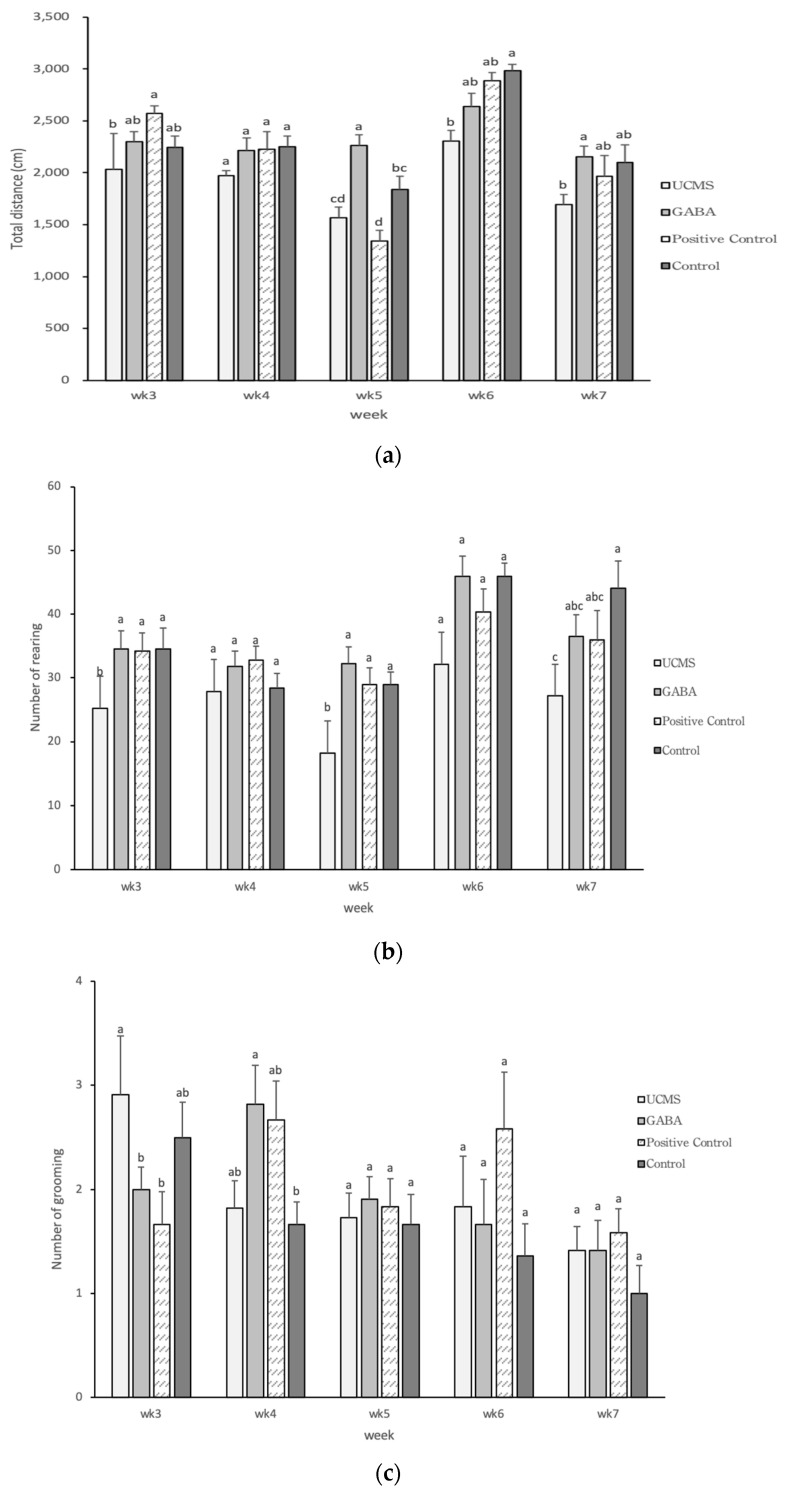

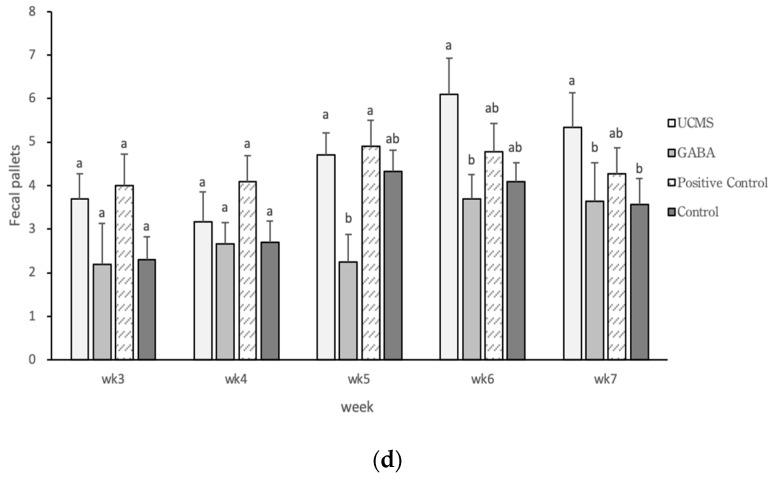
Effect of GABA feed by vegetable soybean on the total distance of mice in open field test (OFT). Data are represented as mean ±SD. (**a**) total travel distance (**b**) number of rearings (**c**) number of grooming episodes (**d**) number of defecations. Group with different letter superscripts are significantly different (*p* < 0.05).

The OFT is used to evaluate an animal response to a novel environment or to stress. Previous studies have indicated that rats and mice of different strains displayed different behaviors due to genetic variations [63,64]. In the present study, the travel distance of the GABA group was higher than that of the UCMS group and was similar to the control group. This observation indicated that feeding mice with the GABA-enriched vegetable soybean seeds could reduce the occurrence of depression (Figure 4a). The willingness of mice to explore the surrounding environment is reflected by the number of rearings and a higher degree of depression can reduce their willingness to explore. At week 5, the number of rearings was significantly higher in the GABA group than in the UCMS group. In addition, the results of the GABA group showed a similar trend to the control group. This finding indicated that the GABA feed could alleviate depressive symptoms, and could even lead to the same degree of emotion as in the control group (Figure 4b). Monteiro et al. [65] showed that C57BL/6 male mice with depression induced by chronic unpredictable stress (CUS) had a significantly higher number of rearings than in the control group, a result similar to that in the present study. Grooming is an important behavior with evolutionary significance and can be observed in many animal taxa. In addition to basic hygiene, combing, and body care, grooming also serves other important functions, including skin stimulation, temperature regulation, social interaction and stress relief. Grooming behavior accounts for 15%–50% of awake time in mice, and it may be triggered by novelty, swimming, pain, and/or predators. Grooming behavior is regulated by multiple brain regions and endogenous factors. The number of grooming episodes is reduced in mouse models with induced depression. At week 4 of the present experiment, the number of grooming episodes was higher in the GABA group than in the UCMS group, indicating that mice in the GABA group were more relaxed (Figure 4c). The number of defecations of mice during an OFT represents the nervousness and anxiety of mice in the exposed environment. In this study, the number of defecations in the GABA group was significantly lower than that in the UCMS group from weeks 5 to 7, indicating that GABA could reduce the degree of depressive symptoms (Figure 4d).

**Figure 5:**
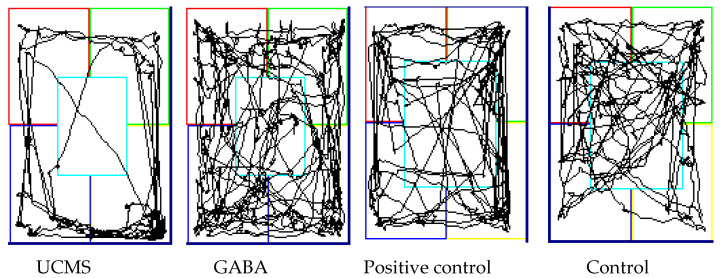
Path map of mice in open field test.

Since rodents naturally display thigmotaxis, the center area of an unfamiliar environment is considered threatening, while the peripheral areas are considered safer [66]. The central area in an unfamiliar environment signifies a threat situation for mice. It was confirmed by animal experiments that in the open space test, an increase in number and time of crossing the central area indicates a decrease in anxiety (Table 2). In the OFT of the present study, the movement trajectories of the mice were recorded (Figure 5). The results showed that the UCMS group spent more time on the periphery and did not pass through the central area, while the trajectories of the GABA, positive control and control groups showed that mice had passed through the central area.

#### 3.3.5. Biochemical Parameter Analyses

**Figure 6:**
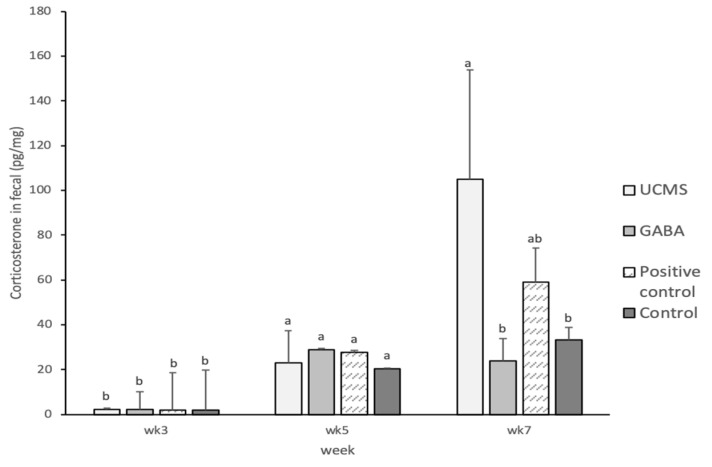
Effect of GABA feed by vegetable soybean on corticosterone content in mice fecal (CORT). Data are represented as mean ±SD. Group with different letter superscripts are significantly different (*p* < 0.05).

It has been clinically proven that the dysregulation of the hypothalamic-pituitary-adrenal (HPA) axis due to stress is related to depression. Animal studies have shown that hormone levels increase in response to stress, which promotes the secretion of CORT or adrenocorticotropin (ACTH) and, in turn, elicit an immune response in the central nervous system. Continuous activation of the HPA axis and excessive hormonal secretions may result in symptoms of depression. Thus, CORT can serve as an indicator of stress [67]. Previous studies have shown that the plasma CORT levels of Swiss mice subjected to four weeks of UCMS induction are significantly higher than that of the control group [54]. In the present study, the fecal CORT levels were not significantly different across the groups before the induction of depression. At week 7, the fecal CORT level in the UCMS group increased to 104.86 pg/mg, which was significantly higher than that in the GABA (23.98 pg/mg) and Control (33.38 pg/mg) groups. The lower fecal CORT level maintained in the GABA group indicated that GABA could alleviate depression (Figure 6).

**Figure 7:**
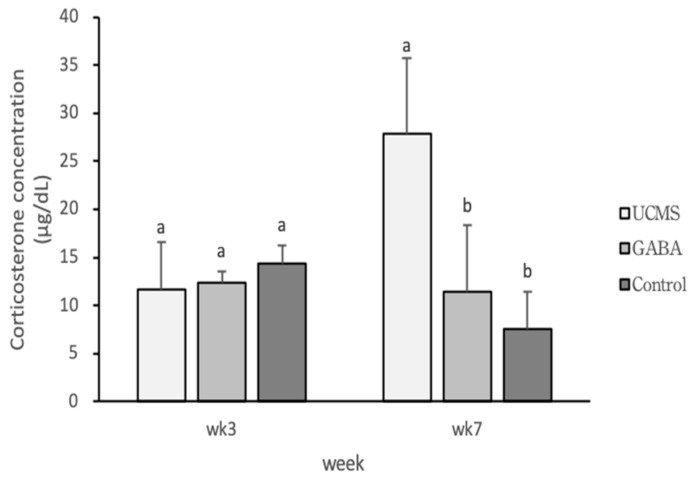
Effect of GABA feed by vegetable soybean on corticosterone content in mice serum. Data are represented as mean ± SD. Group with different letter superscripts are significantly different (*p* < 0.05).

**Figure 8:**
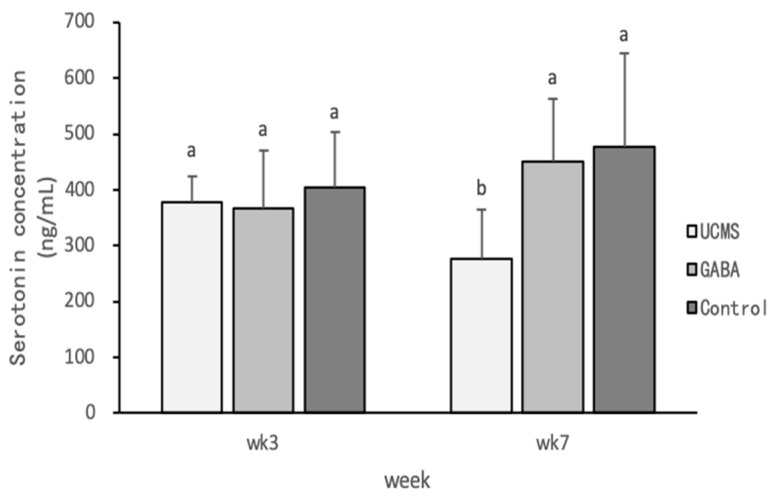
Effect of GABA feed made by vegetable soybean on serotonin content in mice serum. Data are represented as mean ±SD. Group with different letter superscripts are significantly different (*p* < 0.05).

Serotonin is a monoamine neurotransmitter derived from tryptophan. The binding of serotonin to its receptor leads to the activation of metabolic pathways and may affect functions such as appetite, sleep, and depression. Studies have shown that depression is primarily related to serotonin disorders [68]; for example, the patient’s brain may have reduced concentrations of the neurotransmitter serotonin and adrenaline. Therefore, serotonin can be used as an indicator of depression [69,70]. Our experimental results showed that the serum CORT levels also exhibited a consistent trend (Figure 7), demonstrating that GABA could effectively reduce CORT levels in mice. In addition, the serum serotonin and CORT levels showed an opposite trend (Figure 8). It was suggested that the function of serotonin receptors in rodents is weakened after receiving chronic stimulation or high doses of CORT. Hence, depression might lead to a reduced serotonin concentration [71,72].

## 4. Conclusions

GABA has been known as a bioactive component in foods and pharmaceuticals and its various beneficial effects have been well documented. However, the concentration of GABA in common natural food sources is quite low. Consumers prefer natural foods’ efficacious ingredients much more than those externally added to the foods. In this study, GABA content in vegetable soybeans was successfully increased using HPP technology. Furthermore, a UCMS model was employed to induce depression in C57BL/6J male mice and demonstrate that the GABA-enriched vegetable soybeans could significantly alleviate symptoms of depression in mice. The results of fecal CORT, serum CORT, and serum serotonin analyses in mice also suggested that the GABA-enriched vegetable soybeans used in this study could serve as potential supplements for the prevention or control of depression symptoms.

## Figures and Tables

**Table 1:** Influence of high-pressure processing (HPP) treatment and storage on gamma aminobutyric acid (GABA) and L-glutamate.

	Storage Days	Control	200 MPa	400 MPa	600 MPa
GABA (mg/100 g)	1	261.42 ± 3.66 ^a^	436.05 ± 20.57 ^c^	329.55 ± 23.20 ^a^	86.00 ± 6.58 ^b^
	3	241.15 ± 3.93 ^a^	300.32 ± 19.79 ^a^	317.09 ± 28.24 ^a^	70.63 ± 2.96 ^a^
	5	245.1 ± 21.18 ^a^	383.66 ± 0.53 ^b^	288.46 ± 24.97 ^a^	107.14 ± 1.58 ^c^
Glutamate (mg/100 g)	1	63.48 ± 3.38 ^a^	28.29 ± 1.92 ^a^	69.09 ± 11.18 ^a^	275.09 ± 6.61 ^a^
	3	78.58 ± 1.21 ^b^	41.60 ± 0.95 ^b^	85.88 ± 9.15 ^a^	226.28 ± 15.59 ^a^
	5	51.51 ± 5.52 ^a^	41.05 ± 2.01 ^b^	51.45 ± 5.75 ^a^	431.7 ± 44.23 ^b^

Note. Each value is the mean ± SD. (*n* = 3). Different letters indicate significant statistical differences (*p* < 0.05, using Tukey test).

**Table 2:** The distance in zone (%) and time in zone (%) of the mice through the central area in open field test.

	Treatment Group	Week4	Week5	Week6	Week7
Distance in Zone (%)	UCMS	20.32 ± 4.2 ^a^	21.81 ± 4.21 ^a^	14.41 ± 2.72 ^a^	15.54 ± 2.55 ^a^
	Control	22.95 ± 3.18 ^a^	26.85 ± 1.88 ^a^	35.53 ± 5.01 ^b^	26.9 ± 6.77 ^b^
	Positive control	28.02 ± 1.81 ^b^	26.56 ± 5.67 ^a^	35.99 ± 7.37 ^b^	26.21 ± 4.55 ^b^
	GABA	32.8 ± 5.31 ^b^	40.75 ± 2.09 ^b^	31.39 ± 4.27 ^b^	27.52 ± 3.23 ^b^
Time in Zone (%)	UCMS	18.9 ± 4.64 ^a^	19.81 ± 6.45 ^a^	10.93 ± 6.61 ^a^	13.62 ± 10.14 ^a^
	Control	25.79 ± 2.4 ^b^	27.76 ± 4.06 ^ab^	42 ± 8.22 ^b^	37.15 ± 12.64 ^b^
	Positive control	33.88 ± 2.41 ^c^	36.99 ± 2.38 ^bc^	35.18 ± 6.69 ^b^	34.73 ± 6.95 ^b^
	GABA	29.44 ± 1.95 ^c^	41.94 ± 7.14 ^c^	44.71 ± 7.36 ^b^	32.46 ± 4.35 ^b^

Data are represented as mean ± SD. Group with different letter superscripts are significantly different (*p* < 0.05).
